# Skin Diseases and Syphilis

**Published:** 1894-09

**Authors:** 


### SKIN DISEASES AND SYPHILIS.

EDITED BY DR. M. B. HUTCHINS.

### THE RELATIONS OF DERMATOLOGY TO GENERAL MEDICINE.*

Dr. Pye-Smith in his opening remarks called attention to the difficulties which have attended the progress of dermatology, partly due to the tendency on the part of physicians to adhere to the ancient traditions, and to attribute cutaneous affections to hypothetical causes.

One of the hypotheses was that affections of the skin were due to a widespread condition, known as scorbutus or scurvy, and that even now patients sometimes speak of eczema, scabies, or syphilis, as "a breaking out of the scurvy."

Other conditions, again, were ascribed to scrofula or struma, and in a similar manner even the term syphilis was loosely applied, and

*Abstract of address delivered at Dermatological Society of Great Britain and Ireland by Dr. Pye-Smith, F.R.S.

made to account for whatever could not be understood. Long continued observation and experience have proved most of these theories to be erroneous, and have been the means of establishing a *true* etiology. The study of dermatology must begin by accurate observation of anatomical facts, and not until the various anatomical lesions have been recognized, and their microscopical as well as their external characters thoroughly investigated, should a pathological classification be commenced.

In endeavoring to give a forecast of the future of dermatology, he hoped it would never again fall into the hands of empirics, but will always be studied as a branch of scientific medicine; that investigations will be carried out thoroughly, and according to the most recent methods of research; that increased importance will be attached to the characters of local distribution of cutaneous affections, to the action of local infection and to geographical distribution; that in diagnosis the error of multiplying minute distinctions will be avoided; and that, in the treatment of cutaneous affections, external remedies will be used more intelligently, and therefore more confidently.

Referring to the present confused state of nomenclature of dermatology, Dr. Pye-Smith suggested:

1. That every disease should have a single distinctive title, capable of forming an adjective, and, if possible, of Greek or Latin form.

2. That no term used by a writer previous to Willan and Bateman be considered of authority; but that terms used by them and by subsequent writers in first describing a disease be adhered to as far as possible.

3. That new names should be coined only after due consultation, and, if possible, oral discussion.

4. That the number of diseases named after their discoverer should not be added to.—*British Journal of Dermatology*, July, 1894.

---

### ON EXTRAGENITAL SYPHILITIC INFECTION.

Dr. Rudolph Kraefting, Christiania, Norway. (*Norsk Magazin for Laegevidenskaben*, No. 2, 1893.) The writer has made a study of the cases of extragenital syphilitic infection observed in the clinic of that city. From 1867 to 1891, 2,916 cases of syphilis presented 539 cases of extragenital infection, 15.6 per cent. of the total number of the cases. Of these 292 were adults, 61 males and 231 females, and 247 children. Of the males 4.3 per cent., of the females 12.8 per cent., contracted it extragenitally. Since the suppression of official control of syphilis in 1888, genital syphilis has increased, while extragenital infection has decreased. In two hundred and eighty cases the primary lesion was situated as follows:

| | | | | |
|---|---|---|---|---|
| Lips | 142 cases, | 35 males, | 77 females, | 30 children. |
| Gums | 1 " | 1 " | — " | — " |
| Tongue | 11 " | 3 " | 3 " | 5 " |
| Fauces | 58 " | 11 " | 47 " | |
| Mammæ | 58 " | — " | 58 " | |
| Chin | 1 " | — " | 1 " | |
| Forehead | 1 " | — " | 1 " | |
| Scalp | 2 " | 1 " | 1 " | |
| Thigh | 1 " | — " | 1 " | |
| Abdomen | 1 " | 1 " | — " | |
| Fingers | 4 " | 3 " | 1 " | |

Regional glandular swellings often give valuable symptomatological indications on the mode of invasion, even when the traces of the primary lesion are but indistinct. In labial and faucial infection there is nearly always an asymmetric enlargement of the neighboring glands and a resultant deformity of the neck. In chancre of the tonsil induration was often observed, but it is not indispensable. In lesions of the mammary gland induration was always present and diagnosis presented no difficulties. In all the cases, excepting one hundred, there was a clear history as to the method of contagion. In three-fourths of them infection was attributed to contaminated drinking vessels, pipes or cigars, kissing, infection of the nursling by the nurse, or *vice versa*.

He mentions twenty-two cases where there was a veritable epidemic affecting a whole house or family. As to evolution, severe forms seem to be relatively frequent. In adults with the primary sore on the finger or mammæ the disease often assumes a malignant character, with late recurrences. As to the treatment, it differed from the ordinary, in that mercurials were only exceptionally employed while the iodide of potash was used, especially when there was a tendency to chronicity. Tonics were also administered, iron and quinine. In iritis, atropine, vesicatories and leeches to the temples and the iodide were used.—F. H. PRITCHARD, in *Journal of Cutaneous and Genito-Urinary Diseases*, July, 1894.

---

### PSEUDO-CHANCRES.

J. P. Gayon, City of Mexico. (*Gaceta Medica de Mexico.*) The author states that any simple ulceration under the local irritating influence of calomel, a caustic, or even from inflammation of the tissue, may simulate a hard chancre. The site of the lesion exerts a great influence upon the development of induration, for, in the nose and the balano-preputial sulcus, the anatomic disposition of the tissues and the lack of elasticity contribute to produce circumscribed indurations. Any lesion of the superficial layers of the skin or a herpetic eruption in these regions may be complicated with an induration, without any specific element. Any lesion or eruption in the balano-preputial sulcus, is accompanied by more or less induration. Certain syphilides of the mucous membranes take on a certain degree of induration and may simulate a primary sore. This variety being of the papulo-erosive form and from its site, indolence, volume, configuration, evolution and course may so simulate a primary sclerosis that confusion is easy. During the third stage the gummata of the genital organs during ulceration may assume the appearance of the primary sore. They first feel like a foreign body beneath the skin, from their cartilaginous hardness. After a time they approach the skin and, by ulcerating, may resemble either a hard or soft chancre. He concludes as follows:

1. Simple ulcerations of the genital organs by reason of their site or inadequate treatment may simulate a hard chancre.

2. Soft chancres, either from these same causes or from developing in a syphilitic organism, may also give rise to the same confusion.

3. Certain syphilides of the secondary and syphilomata of the tertiary period may perfectly assume the characteristics of the initial lesion of syphilis.—F. H. PRITCHARD, in *Journal of Cutaneous and Genito-Urinary Diseases*, July, 1894.

### BILATERAL INGUINAL BUBOES FROM PEDICULOSIS PUBIS.

Dr. R. Kraefting, of Christiania. (*Norsk Magazin for Laegevidenskaben,* No. 1, 1892.) The writer reports the case of a young man of twenty-three years old, who came under treatment for a bubo in each groin but who presented neither a soft chancre, balanitis nor a gonorrhea. Examination revealed an enormous quantity of pediculi pubis, which had given rise to eczematous patches above the pubic symphysis. A certain quantity of pus was extracted from the right bubo but no micro-organism could be discovered, either by the microscope, culture or inoculation. The leucocytes stained imperfectly—sign of degeneration. Otherwise, they pursued the course of ordinary non-virulent buboes after soft chancre and were but slightly inflamed. The pus was of a grayish color, and herein differed from the chocolate-brown pus of virulent buboes, and the yellowish-green pus of ordinary abscesses. He leaves it undecided whether the suppuration here was due to products excreted by the pediculi or to an irritation of the skin from their presence. No microbes could, however, be made out with the usual methods of investigation. Under a compressing bandage the pus was, in a great measure, absorbed as with non-virulent buboes after soft chancre.—F. H. PRITCHARD, in *Journal of Cutaneous and Genito-Urinary Diseases,* July, 1894.

---

### MIXED CHANCRE.

Balzer (*La Med. Mod.,* 6 May, 1893) says the knowledge of the difference between ulcus molle (chancroid) and ulcus syphiliticum (true chancre) guides to the distinction of the mixed chancre. A patient may be simultaneously infected with the two poisons or one infection may follow the other.

The first may occur:

1. From a mixed chancre.

2. The infected person has a soft chancre along with lesions of syphilis.

3. The infecting person has a soft chancre while her syphilis is either latent or yet communicable.

The infection in turn may occur as follows:

1. A person having soft chancre acquires syphilis.

2. A syphilitic acquire soft chancre.

The method of origin is important for clinical observation. With simultaneous infection the soft chancre appears first, it becomes indurated by the fourteenth to twentieth day, sometimes when the ulcer is entirely healed. It is useful if one sees, in the immediate neighborhood of the mixed lesion, small, soft chancres. If one finds with those a group of enlarged (inguinal) glands, connected with a suppurative bubo, the diagnosis of *ulcus molle mixtum* is easy.—*M. für Prak. Derm.*, XVIII., No. 7.

---

### THE RELATION OF ECZEMA TO THE MUCOUS MEMBRANES.

Dr. von Sehlen, of Hannover, concludes an article upon this subject (*M. für P. Derm.* xix., No. 1) as follows:

1. Chronic eczema of the skin can encroach upon the adjacent mucous membrane and produce apparently the same disease.

2. Eczema of the lips, catarrh of the external auditory canal, eczema of the lids, and a certain form of conjunctivitis are to be considered as special localizations of the eczematous process, and treated accordingly.

3. Certain inflammatory conditions of the anal mucous membrane, and of the genitals of both sexes appear to stand in close, if not in "causal," relation to eczema of the skin.

---

### INTERMISSIONS IN TUBERCULAR SKIN DISEASES.

Hallopeau (*Journ. des. Mal. Cut. et Syph.*, 1893, p. 726) says that long or short suspensions of the process may occur in nearly all forms of skin tuberculosis. This is more frequent in lichen scrophulosorum, and those cases depending upon tubercular disease of the subjacent tissue. In lupus also a long continued suspension of activity may occur under appropriate treatment. He mentions one case where a lupus scar remained quiescent for forty years. One can never speak of a permanent cure of this disease.—*M. für Prak. Derm.*, xix., No. 1.

### EPITHELIOMA OF THE UPPER LIP FOLLOWING LUPUS.

Dubreuilh (*Arch. Clin. de Bordeaux,* 1893, No. 12) gives the clinical history of this case. Patient was forty-three years old. The very hard, sharply circumscribed new growth began on the border of the lupous part and invaded the tissues not cicatricially changed.—*M. für Prak. Derm.*, xix., No. 1.

